# Value of multiparametric magnetic resonance imaging in distinguishing sinonasal lymphoma from sinonasal carcinoma: a case control study

**DOI:** 10.1186/s12880-024-01366-6

**Published:** 2024-07-24

**Authors:** Chong Liu, Ye Wang, Duo Zhang, Jin Zhou, Yan Wu, Ying Guo, Rui-Chao Liu, Jin-E Xu

**Affiliations:** 1 Department of Medical Imaging, Central Hospital, Baoding No.1, 320 Changcheng North Street, Lianchi District, Baoding, Hebei Province China; 2 Department of Medical Imaging, General Hospital of North China Petroleum Administration Bureau, Renqiu city Huizhan road, Cangzhou, Hebei Province China; 3 General Surgery Department, Central Hospital, Baoding No.1, 320 Changcheng North Street, Lianchi District, Baoding, Hebei Province China

**Keywords:** Magnetic resonance imaging, Sinonasal, Lymphoma, Carcinoma

## Abstract

**Background:**

The study aimed to evaluate the diagnostic efficacy of dynamic contrast-enhanced magnetic resonance imaging (DCE-MRI) and diffusion-weighted imaging (DWI) parameters in distinguishing sinonasal lymphoma from sinonasal carcinoma.

**Methods:**

Forty-two participants with histologically confirmed sinonasal lymphomas and fifty-two cases of sinonasal carcinoma underwent imaging with a 3.0T MRI scanner. DCE-MRI and DWI were conducted, and various parameters including type of time-intensity curve(TIC), time to peak, peak enhancement, peak contrast enhancement, washout rate, apparent diffusion coefficient (ADC), and relative ADC were measured. Binary logistic regression and receiver operating characteristic (ROC) curve analysis were employed to assess the diagnostic capability of individual and combined indices for differentiating nasal sinus lymphoma from nasal sinus carcinoma.

**Results:**

Sinonasal lymphoma predominantly exhibited type II TIC(*n* = 20), whereas sinonasal carcinoma predominantly exhibited type III TIC(*n* = 23). Significant differences were observed in all parameters except washout ratio (*p* < 0.05), and ADC value emerged as the most reliable diagnostic tool in single parameter. Combined DCE-MRI parameters demonstrated superior diagnostic efficacy compared to individual parameters, with the highest efficiency (area under curve = 0.945) achieved when combining all parameters of DCE-MRI and DWI.

**Conclusions:**

Multiparametric evaluation involving contrast-enhanced dynamic MRI and DWI holds considerable diagnostic value in distinguishing sinonasal lymphoma from sinonasal carcinoma.

## Introduction

Due to the complex anatomical structure of the nasal cavity and paranasal sinus, a wide range of benign and malignant tumors may arise. Benign tumors predominantly consist of inflammatory polyps, while malignant tumors primarily encompass various forms of malignancies [1]. When compared to other malignancies affecting the head and neck, sinonasal malignancies stand out as relatively infrequent, accounting for approximately 3–5% of head and neck malignancies and 0.2–0.8% of systemic malignancies [2–4]. Sinonasal lymphoma (SL) constitutes a heterogeneous hematopoietic malignancy characterized by the abnormal proliferation of mature lymphoid cells or their precursors, subclassified into Hodgkin’s lymphoma (HL) and Non-Hodgkin’s lymphoma (NHL) [5], which representing roughly 6–13% of all extranodal head and neck lymphomas [6]. Lymphoma represents a prevalent subtype of sinonasal malignancies, distinguished by its non-epithelial nature. Conversely, sinonasal carcinoma (SC) emerges as a primary malignancy in the sinonasal region, with squamous cell carcinoma (SCC) being its most prevalent epithelial subtype. It occurs more frequently than SL, representing 40–50% of all nasal malignant tumors [6].

Distinguishing SL from SC poses a diagnostic challenge due to several factors. Because the nasal cavity and sinuses have a confined anatomical space, these lesions often grow silently, and their clinical presentations such as nasal obstruction, local pain, and epistaxis lack specificity [6]. Moreover, despite the feasibility of endoscopic incisional biopsy in the sinonasal area, diagnostic accuracy remains uncertain due to sampling limitations, with a sensitivity of only 43.7% reported [7]. Furthermore, divergent treatment modalities exist for SL and SC, with lymphoma patients typically receiving nonsurgical therapies such as chemotherapy or radiotherapy, while surgical resection remains the preferred approach for SC patients, with adjuvant radiotherapy or chemotherapy considered selectively [6, 7]. Therefore, accurate preoperative identification of SL and SC can improve patient management and prognosis.

Conventional MRI findings, such as signal intensity homogeneity and involvement of adjacent structures, lack specificity, rendering SL and SC differentiation challenging [8]. Consequently, novel imaging modalities are imperative for improved diagnosis. Recent investigations focusing on the head and neck regions have demonstrated the utility of functional MRI techniques such as diffusion-weighted imaging(DWI) and dynamic contrast-enhanced magnetic resonance imaging (DCE-MRI) in enhancing tissue characterization and understanding physiological processes associated with tumor diagnosis [9–15]. Parameters such as the apparent diffusion coefficient (ADC) derived from DWI and time-intensity curves (TIC) obtained from DCE-MRI provide more reliable basis for distinguishing benign and malignant tumors of head and neck, including SC, SL and benign/malignant tumors of salivary gland, and show promise in predicting tumor molecular phenotype, monitoring treatment response and evaluating patients’ post-treatment changes [6, 16–20].

However, there is a paucity of literature addressing the value of multiparametric MRI in distinguishing SL from SC, with most studies concentrating on clinical symptoms, conventional imaging characteristics, or individual parameters such as DWI or DCE-MRI for differentiating between the two types of lesions [8, 10, 21]. This study aims to ascertain whether multiparametric MRI, incorporating DCE-MRI and DWI, can effectively differentiate between SL and SC.

## Materials and methods

### Patients

The study was conducted in accordance with the Declaration of Helsinki and the agreement was approved by our Institutional Ethics Committee (fast [2022]046), with written informed consent waived due to the retrospective study. We retrospectively reviewed the medical records and imaging results of patients with pathologically confirmed SL, SC between 2014 and 2023. Patients without prior biopsy, surgery or treatment, as well as those successfully examined with conventional MRI imaging, DWI and DCE-MRI were included in the study. Exclusion criteria were as follows: (1) recurrent lesions; (2) history of craniofacial trauma or nasal surgery; (3) absence of DWI, DCE-MRI; and (4) presence of distant lymph node metastases. There were 52 patients with SC, comprising 11 females and 41 males, and their average age was 64.1 years. Additionally, there were 42 patients with SL, including 12 females and 30 males, and their average age was 62.2 years.

### MRI examinations

All images were performed using a 3.0 T device (Achieva TX; Philips Healthcare, Best, Netherlands) with a 16 channel neurovascular coil. The scanning sequence consisted of (1) axial T1 weighted spin echo (TR/TE/NEX 400 ~ 675ms/10-18ms/4, 4-mm slice thickness, 1 mm gap, 244 × 188 matrix, 220 mm×105 mm FOV images; (2) axial/sagittal/coronal T2-weighted spin-echo images with fat suppression (TR/TE/NEX 2500 ~ 3500ms/90 ~ 130ms/4, 4 mm slice thickness, 1-mm gap, 244 × 149 matrix, 220 mm×105 mm FOV images); (3) DWI (TR/TE 4156ms/55ms; slice thickness of 4 mm; gap of 0.4 mm; matrix size 116 × 114; b value, 0 and 800s/mm^2^); and (4) DCE-MRI using the eTHRIVE sequence (TR/TE 3.3ms/1.62ms, flip angle 10°, 220 × 218 matrix, 1 mm slice thickness). First, a scan was performed prior to contrast agent injection; then, a continuous scan was performed 10 times after injection. Gadopentetate Dimeglumine injection ( Bayer Healthcare Pharmaceuticals, Germany) was administered intravenously at a rate of 2mL/s (total dose, 0.1mmol/kg of body weight), followed by a flush of 10mL of saline solution.

### Image analysis

ADC value was obtained by two evaluators with 9 and 10 years of expertise in head and neck imaging, respectively, independently and blind at Philips post-processing station according to DWI post-processing. Refer to the measurement method of the previous study [16], the ROIs of ADC value were divided into three parts: the layer with the largest tumor diamete and its adjacent up/down sections. The conformal ROI was manually drawn along the edge of the lesion in each part to obtain three ADC values, avoiding areas of hemorrhage and necrosis. The uninvolved lateral pterygoid muscle at the same level was selected, and the circular ROI (10–20 mm^2^) was manually drawn to obtain the relative ADC (rADC), that is, the ratio of the ADC value of the lesion to the ADC value of the muscle. The ADC values and rADC values obtained from these three sets of ROIs were averaged, and the final ADC value and rADC value of each surveyor were the average values of the three levels. The κ statistic [6] was used to evaluate the reliability between the scores of the two surveyors. κ values are interpreted as follows: 0.00 ~ 0.20 indicates slight agreement, 0.21 ~ 0.40 indicates general agreement, 0.41 ~ 0.60 indicates moderate agreement, 0.61 ~ 0.80 indicates good agreement, and 0.81 ~ 1.00 indicates very good agreement.

**Fig. 1:**
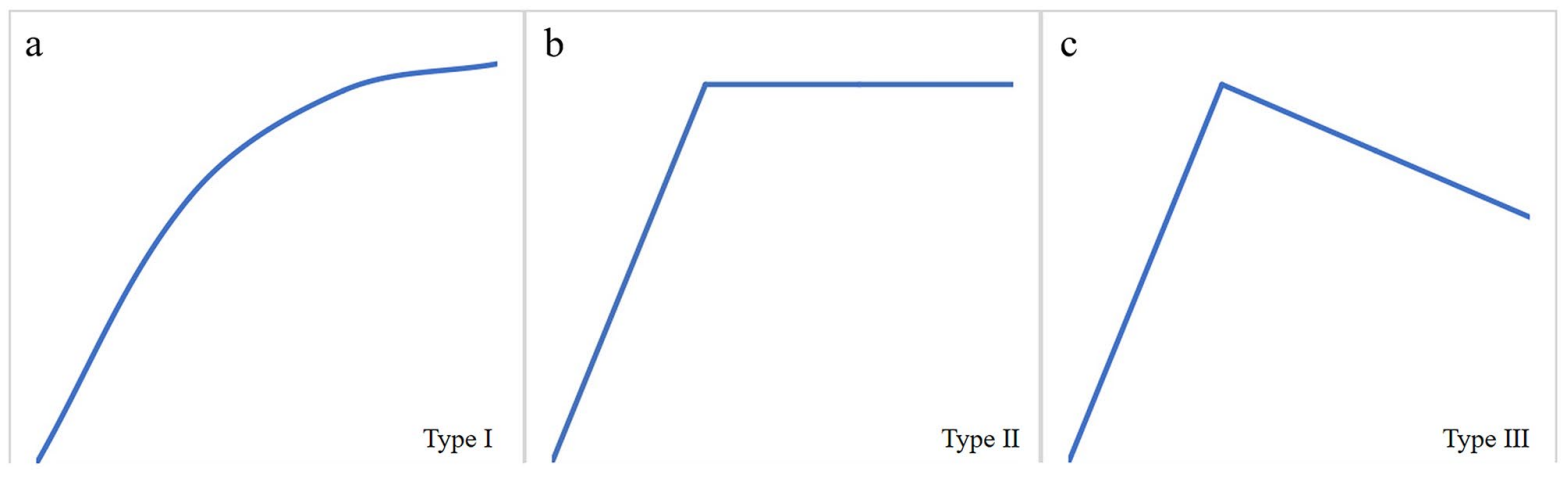
Time intensity curve (TIC) analysis and classification. The TICs were classified into three types. (**a**). Type I: uptrend type—continuous ascending curve without an EP; (**b**). Type II: platform type—rapidly ascending to EP followed by a plateau, with a WR < 10%; (**c**). Type III: wash-out type—rapidly ascending to EP followed by a descending curve, WR > 10%.

DCE-MRI analysis was performed using the MEAN-CURVE software package with internal parsing. ROI for each multiphase image was manually delineated. Two radiologists, blinded to the pathological types of lesions, independently observed each set of dynamic enhanced images and selected the phase and slice with maximum enhancement. In cases of discordance, consensus was reached after consultation.Then a circular ROI with an area of about 10–20 mm^2^ was placed in the area with the largest enhancement. The software automatically generated TIC and related data, such as pre-enhancement signal intensity (SI1) and final signal intensity (SI10). Calculations of relevant parameters, including peak enhancement (EP), time to peak (TTP), maximum contrast enhancement ratio (MCER), and washout ratio (WR) were performed. EP represented the highest signal intensity occurring at TTP throughout the entire dynamic enhancement process. The formulas for calculating MCER and WR were as follows: MCER = (EP - SI1)/SI1; WR = (EP - SI10)/(EP - SI1). TIC was divided into three categories: Type I: uptrend-continuous ascending curve without EP; Type II: platform type, rapid ascent to EP followed by a plateau with WR < 10%; Type III: washout from the injection rapidly up to the EP and then back down again, WR > 10% (Fig. 1) [16].

### Statistical analysis

Chi square test was used for categorical variables. Continuous variables were compared using student’s t test or mannewhitney U test (if the data did not conform to normal distribution), respectively. For all statistical analyses, *P* < 0.05 was considered statistically significant. A receiver operational characteristic (ROC) curve analysis system was used to determine the diagnostic ability of a single parameter set. The diagnostic ability of the composite endpoints was determined using binary logistic regression and ROC curve analysis. The diagnostic performance of each curve was compared by Delong test, and *P* < 0.05 was statistically significant.

## Results

Following exclusions, the analysis comprised 52 patients with SC, including 45 non-keratinizing SC cases and 7 keratinizing SC cases, and 42 patients with SL, including 24 with NK/T lymphoma, 15 with diffuse large B-cell lymphoma (DLBCL), and 2 with Burkitt’s lymphoma.

The parameters ADC and rADC value exhibited weighted κ values 0.919 and 0.879, respectively, indicating substantial interrater agreement between SL and SC across all metrics, irrespective of DCE-MR or DWI.

**Fig. 2:**
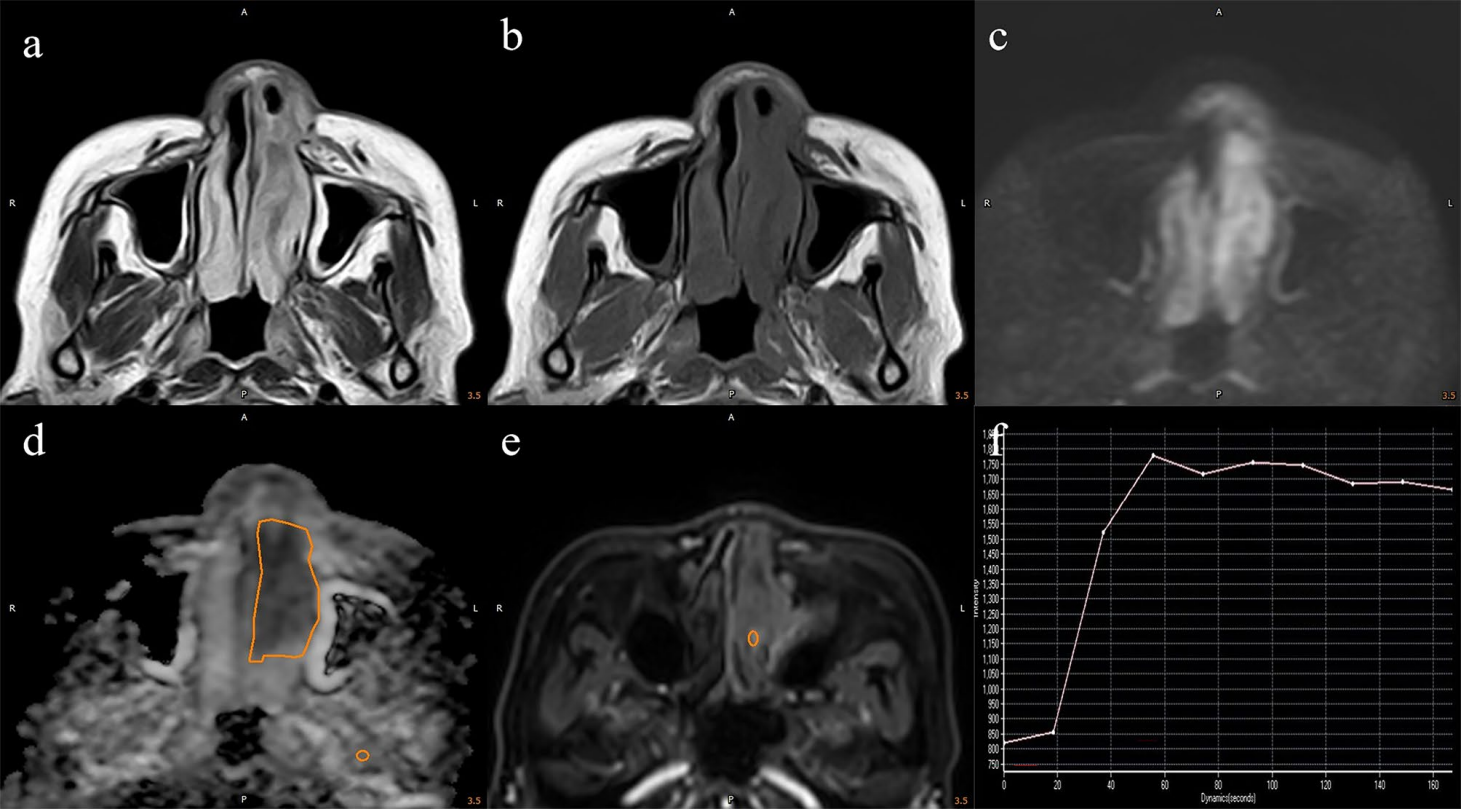
A 57-year-old female patient with sinonasal lymphoma, presenting in the left nasal cavity with relatively uniform signals of slightly high T2WI (**a**) and slightly low T1WI (**b**). The DWI (**c**) demonstrates high signal intensity, while the ADC map (**d**) shows decreased signal. The yellow lines in images **d** and **e** represent the region of interest (ROI) measurement method. Image **f** displays a type II time-intensity curve (TIC)

**Fig. 3:**
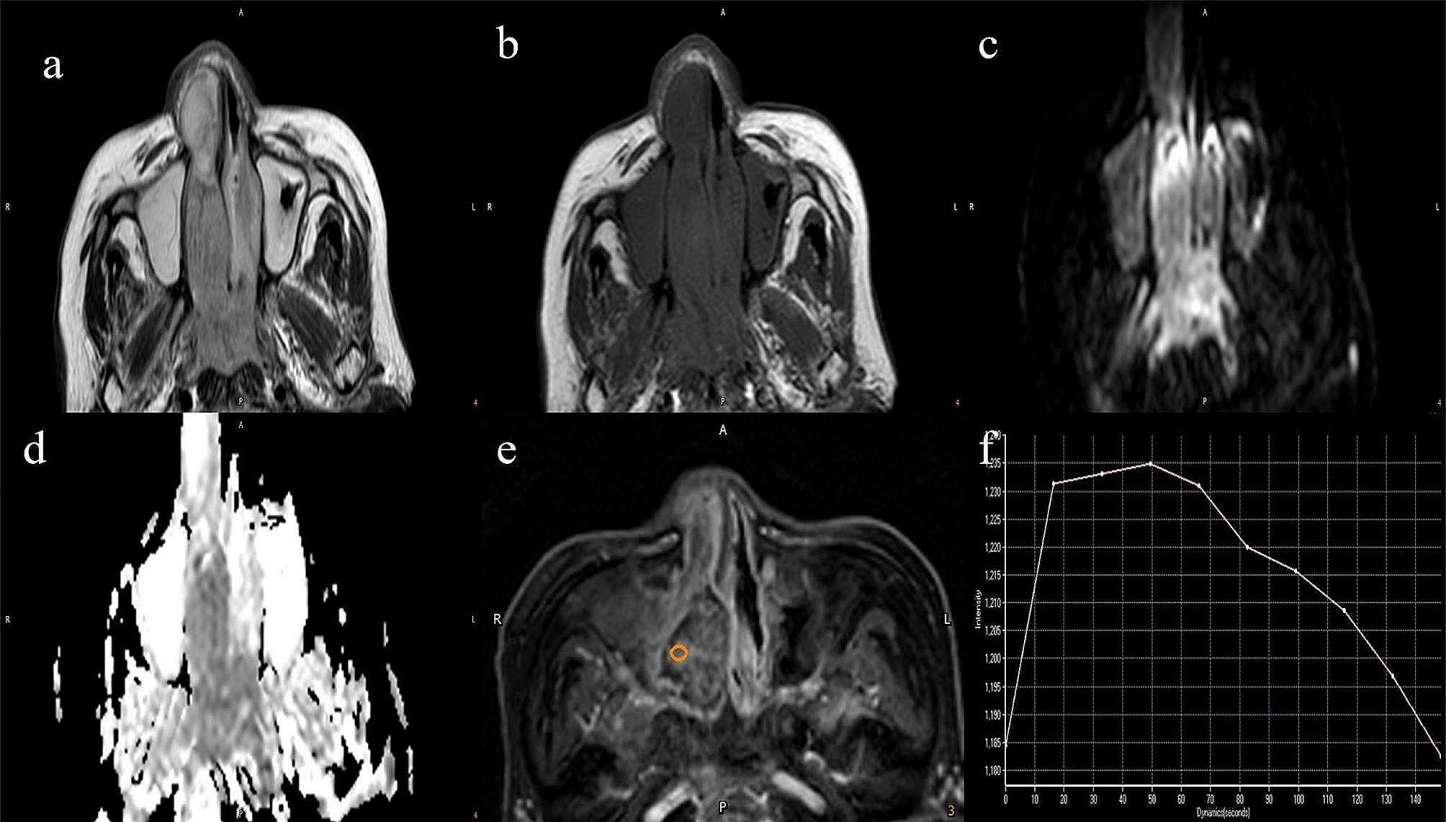
An 82-year-old female patient with sinonasal carcinoma, demonstrated heterogenous signals in the right nasal cavity, nasal cavity, nasopharyngeal cavity, and maxillary sinus with slightly high T2WI (**a**) and slightly low T1WI (**b**). The DWI (**c**) demonstrates high signal intensity, while the ADC map (**d**) shows decreased signal. The measurement method of ROI is the same as Fig. 2. Image e shows heterogeneous enhancement of lesion. Image **f** displays a type III TIC

Table 1 presents the demographic disparities and the dispersal of TIC types among SC and SL. No notable statistical variance was observed in the distribution of lesions concerning age and gender between both groups. Type III was the most prevalent (44.2%) in the SC group, whereas in the SL group, types II; (47.6%) and I (38.0%) predominated. The distribution of TIC types significantly differed between the SC and SL groups (χ^2^ = 9.767, *P* = 0.008). Figures 2 and 3 illustrate typical cases of SL and SC, as well as the method for drawing the ROI.

**Table 1:** Demographic and TIC type difference between SC and SL

Characteristics	SC	SL	χ^2^/t	*P*
Age, mean (± SD)	64.1 (± 7.9)	62.2 (± 7.5)	1.183	0.240
Gender, No. (%)			0.692	0.406
Male	41(78.8)	30(71.4)		
Female	11(21.2)	12(28.6)		
TIC tyeps No. (%)			9.767	0.008
I	13(25.0)	16(38.0)		
II	16(30.8)	20(47.6)		
III	23(44.2)	6(14.2)		

**Fig. 4:**
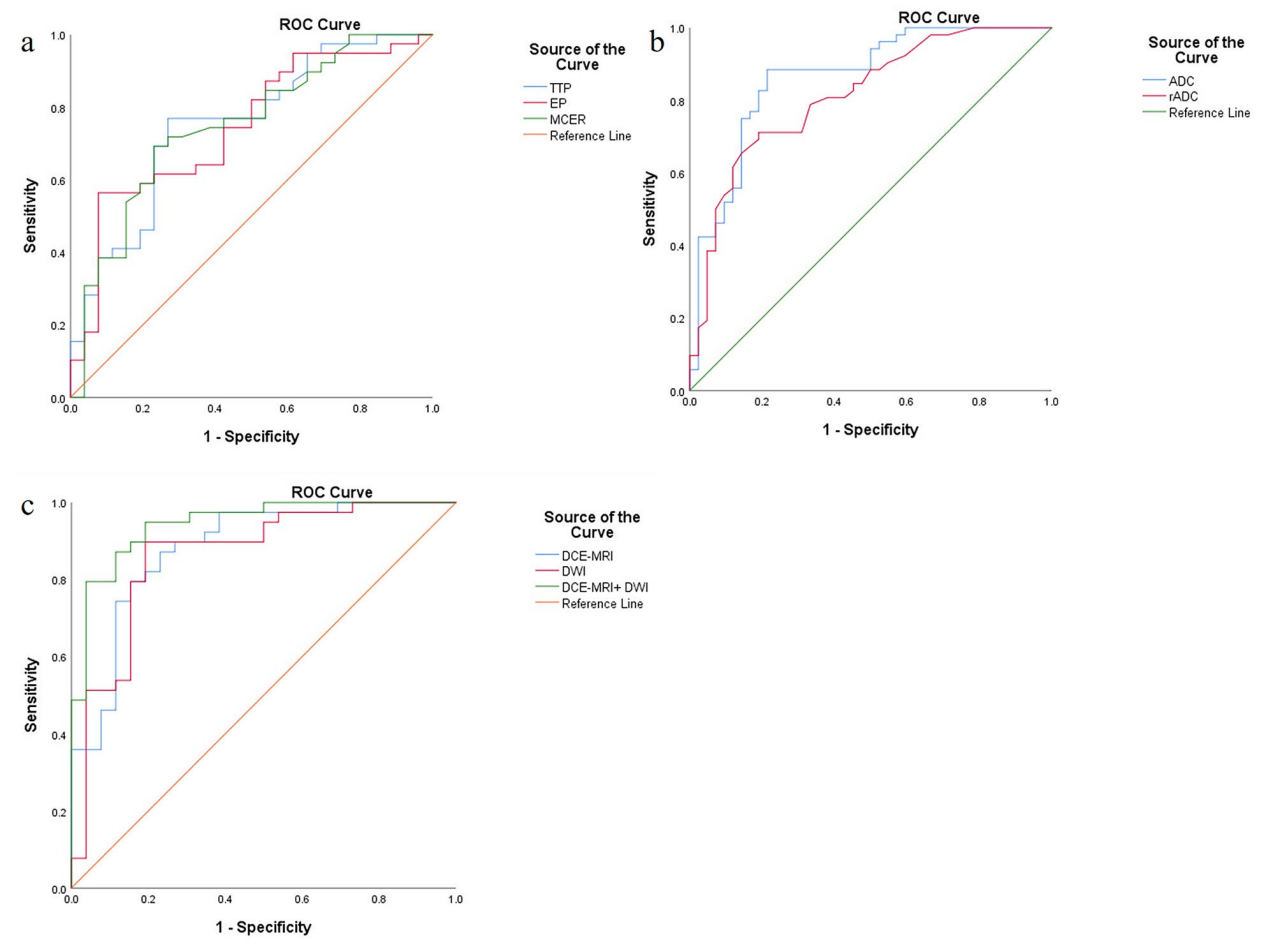
ROC for differentiating SC from SL with (**a**) TTP, EP and MCER; (**b**) ADC value and rADC value; (**c**) DCE-MRI parameters (TTP, MCER and EP), DWI parameters (ADC value and rADC value), and the combination of DCE-MRI and DWI parameters

Significant discrepancies were noted in TTP, EP, MCER, ADC value, and rADC value between the two groups, while WR did not exhibit disparity.The results of a detailed comparison of DWI and DCE-MRI between SC and SL groups are shown in Table 2. Compared to the SC group, the SL group demonstrated prolonged TTP alongside lower EP, MCER, ADC value and rADC value. In terms of individual parameters, the highest area under the curve (AUC) score of 0.863 was observed for ADC value, followed by rADC value (AUC = 0.813), TTP (AUC = 0.753), MCER (AUC = 0.749), and EP (AUC = 0.745). ADC value also exhibited the highest positive, negative, and overall accuracy rates. Detailed diagnostic characteristics for each parameter distinguishing SC from SL are outlined in Table 3 and depicted in Fig. 4a and b.

This study aimed to explore the diagnostic efficacy of combining DCE-MRI parameters (TTP, MCER, EP), DWI parameters (ADC value and rADC value), and the amalgamation of DCE-MRI and DWI parameters (TTP, MCER, EP, ADC value and rADC value) for discriminating between SC and SL (Fig. 4c). The AUCs for these combinations were 0.882, 0.863, and 0.945, respectively, with DCE-MRI combined with DWI exhibiting the most favorable diagnostic capabilities (*P* < 0.05) (Table 3).

**Table 2:** Differences in DWI and DCE-MRI parameters between SC and SL

	TTP(s)	EP	MCER	WR	ADC (× 10^− 6^ mm^2^/s)	rADC
SC	75.70 ± 10.30	948.12 ± 221.81	1.99 ± 0.49	0.21 ± 0.12	1092.69 ± 140.13	0.98 ± 0.30
SL	87.40 ± 13.98	769.18 ± 151.69	1.55 ± 0.59	0.18 ± 0.12	859.61 ± 181.35	0.71 ± 0.19
P	0.000	0.000	0.002	0.513	0.000	0.000

DWI, diffusion-weighted imaging; DCE, dynamic contrast-enhanced; TTP, time to peak; EP, enhancement peak; MCER, maximum contrast enhancement ratio; ADC, apparent diffusion coefficient; rADC, relative ADC

**Table 3:** The diagnostic value of DWI and DCE-MRI parameters in SC and SL

	AUC	Cutoff value	Sensitivity(%)	Specificity(%)	Youden’s index	PPV (%)	NPV (%)	Accuracy (%)	P*
TTP	0.753	76.92	0.769	0.731	0.500	0.811	0.679	0.754	< 0.001
EP	0.745	855.365	0.546	0.923	0.487	0.917	0.585	0.708	< 0.001
MCER	0.749	1.905	0.692	0.769	0.416	0.818	0.625	0.723	< 0.001
ADC	0.863	1011.135 × 10^− 6^ mm^2^/s	0.885	0.786	0.671	0.836	0.846	0.840	0.011
rADC	0.813	0.825	0.712	0.810	0.522	0.822	0.694	0.755	0.005
DCE-MRI	0.882	0.607	0.872	0.769	0.641	0.850	0.800	0.831	0.046
DWI	0.863	0.578	0.923	0.786	0.705	0.842	0.892	0.862	0.011
DCE-MRI + DWI	0.945	-	0.872	0.885	0.757	0.919	0.821	0.877	-

*The *P* value represents the specific result of the Delong test, which compares the combined model (DCE-MRI + DWI) against other models. AUC, area under the curve; DCE-MRI, dynamic contrast-enhanced magnetic resonance imaging; DWI, dynamic weighted imaging; EP, enhancement peak; TTP, time to peak; MCER, maximum contrast enhancement ratio; ADC, apparent diffusion coefficient; rADC, relative ADC. PPV, positive predictive value; NPV, negative predictive value

## Discussion

DCE-MRI and DWI were able to detect differences between SL and SC. The TTP, EP, MCER, ADC value, and rADC value were significantly different from one another. WR, on the other hand, was not significantly different between the two groups. Furthermore, ADC value could be a promising parameter for distinguishing SL from SC. Notably, the amalgamation of all DCE-MRI parameters exhibited a commendable diagnostic efficacy.

DCE-MRI serves as a valuable tool for quantifying microvasculature dynamics, elucidating the physiological characteristics of lesions upon administration of contrast medium [22]. Moreover, literature suggests a correlation between DCE-MRI parameters and key aspects of tumor biology such as angiogenesis, microvessel density (MVD), and proliferating cell nuclear antigen (PCNA) expression [23, 24]. During the initial stages of dynamic scanning, the contrast agent predominantly localizes within the vascular compartment. With an increase in MVD, the rate of signal alteration escalates, leading to a decrease in peak time. Towards the conclusion of the dynamic imaging session, signal alterations predominantly reflect changes in blood vessel and extracellular space (EES) permeability. The diverse performance of tumors can be attributed to the interplay between EES volume and contrast agent kinetics within the EES [16]. Pathologically, our observations are based on the fact that SC has an abundant blood supply, incomplete capillary endothelial cells, and high permeability. Moreover, in the differential study of lymphoma and adenocarcinoma involving other sites, adenocarcinoma has larger EES and larger ve (volume fraction of contrast agent transferred from blood vessels to EES) than lymphoma [25, 26]. This means that the SC enters and exits the contrast medium more quickly, resulting in a higher EP and a shorter duration. This results in most SC having TIC type III, shorter TTP, and higher EP and MCER values. However, SL has high cell density, small microvessels, complete capillary endothelium and poor permeability. This results in most of its TIC type II images with longer TTP and lower EP and MCER values. Our observations are in agreement with previous studies [10] in which the majority of malignant lymphoma(ML) exhibit longer TTP (90–180 s) than SC (TTP 60–105 s). More recently, a number of investigators using pseudo-continuous arterial spin labeling have demonstrated that the average blood flow velocity of ML is lower than that of patients with SC [27]. In addition, Lee et al. [9]. reported that the average plasma volume (which is a function of blood volume) is higher in SC compared with ML, although statistical significance was not attained. Which demonstrated a lower degree of ML tumor perfusion compared to SC. The contrast agent also moves in and out slower, the EP is lower, and takes longer. The present results, showing that different tumor types produce significant differences in TIC, TTP, EP and MCER.

SL has also been observed to exhibit lower ADC value than SC in the sinonasal region [6, 12]. In agreement with previous studies, in our study, mean ADC value of SL is lower than SC. ADC value in DWI have been reported to correlate strongly with cellularity [22]. Lymphomas, with a high cell density, have also been shown to have significantly increased cell proliferation and nuclear volume with decreased extracellular matrix and molecular diffusion space, leading to a marked reduction in the degree of diffusion of the water molecules. Simultaneously, the best threshold value of 1011.135 × 10^− 6^ mm^2^/s was demonstrated by ADC value in our study, and the AUC, sensitivity, specificity, and accuracy were 0.863, 0.885, 0.786 and 0.840, respectively. The median AUC (approximately 0.7) for TTP, EP, and MCER was less than that for ADC or rADC. To some degree, these results indicate that DWI is more effective for diagnosis than DCE-MRI.

Presently, an increasing body of literature underscores the utility of DCE-MRI and DWI in the diagnosis of head and neck pathology. Sumi et al. [11] demonstrated enhanced diagnostic accuracy with the combination of DWI and DCE-MRI in differentiating head and neck tumors. Our study aimed to compare the diagnostic performance of these two MRI modalities. Stepwise logistic regression analysis identified TTP, EP, MCER and ADC as the most discriminatory endpoints for diagnosis, reflecting the degree of lesion perfusion and disease distribution. Our results indicate that DWI combined with DCE-MRI is more accurate at discriminating SL and SC than each parameter alone, achieving an accuracy of 87.7%. To the best of our knowledge, there are very few local or international studies that use DWI or DCE-MRI to differentiate SL from SC. This study is the first to use multiparametric imaging to differentiate between SL and SC. It not only lays the groundwork for future research but also provides clinical physicians with abundant case data, so that they could receive more benefits.

The findings of our study should be interpreted within the context of several inherent limitations. Primarily, our investigation did not differentiate between various subtypes of SC (keratinized and non-keratinized) and SL (NK/T, DLBCL), limiting the comprehensiveness of our analysis. Future endeavors should aim to expand the sample size, encompassing a broader spectrum of diseases, and facilitating comparative diagnostic assessments across different pathologies, thereby furnishing clinicians with more comprehensive insights to inform clinical decision-making. Moreover, intraoperative lesion removal in fragmentary form precluded precise correlation with MRI findings, impeding accurate assessment of lesion characteristics. Additionally, the application of DCE-MRI was confined to semiquantitative analysis, warranting further exploration to elucidate its full potential in this domain. Lastly, the retrospective nature of our study underscores the imperative for prospective investigations to validate our findings in real-time clinical settings and enhance the robustness of our conclusions.

## Conclusions

We conclude that DCE-MRI and DWI can be used for the diagnosis and differentiation of SL and SC, and that TIC, TTP, EP, MCER, ADC value, and rADC value are useful for the differential diagnosis. In summary, the combination of DWI and DCE-MRI is superior to either of the two methods, which can greatly increase the diagnostic accuracy.

## Data Availability

The datasets analyzed in this study are available from the corresponding author on request.

## Abbreviations

DCE-MRI: Contrast-enhanced magnetic resonance imaging
DWI: Diffusion weighted imaging
ADC: Apparent diffusion coefficient
ROC: Receiver operating characteristic
SL: Sinonasal lymphoma
HL: Hodgkin’s Lymphoma
NHL: Non-Hodgkin’s lymphoma
SC: Sinonasal carcinoma
SCC: Squamous cell carcinoma
TIC: Time-intensity curves
ROI: Region of interest
rADC: Relative ADC
EP: Peak enhancement
TTP: Time to peak
MCER: Maximum contrast enhancement ratio
WR: Washout ratio
DLBCL: Diffuse large B-cell lymphoma
AUC: Area under the curve
MVD: Microvessel density
PCNA: Proliferating cell nuclear antigen
EES: Extracellular space
ML: Malignant lymphoma
